# The relationship between servant leadership and followers’ pro-social rule-breaking behavior: a study based on Chinese selected candidates

**DOI:** 10.3389/fpsyg.2025.1588127

**Published:** 2025-11-13

**Authors:** Zhizhong Hu, Honglin Zhang, Xiaoling He, Yingchun Han

**Affiliations:** 1 School of Public Policy and Management, Nanchang University, Nanchang, Jiangxi, China; 2 School of Marxism, Nanchang University, Nanchang, China; 3 School of Marxism, University of Electronic Science and Technology of China, Chengdu, China

**Keywords:** servant leadership, work autonomy, risk-taking willingness, public service motivation (PSM), pro-social rule-breaking (PSRB) behavior

## Abstract

**Objective:**

This study explores the relationships among servant leadership, work autonomy, risk-taking willingness, public service motivation (PSM), and pro-social rule-breaking (PSRB), focusing on their underlying psychological mechanisms.

**Methods:**

A survey was conducted among selected cadres using electronic questionnaires. The questionnaire included demographic information, *the Servant Leadership Scale*, *the Risk-Taking Willingness Scale*, *the Work Autonomy Scale*, *the PSM Scale*, and *the PSRB Scale.* A total of 679 valid responses were collected. Descriptive analyses and moderated mediation tests were performed using AMOS, SPSS, and the PROCESS macro.

**Results:**

Servant leadership showed a significant positive correlation with PSRB. Mediation analysis further indicated that work autonomy and risk-taking willingness exerted significant mediating effects. Servant leadership positively predicted work autonomy, which in turn predicted PSRB. Servant leadership negatively predicted risk-taking willingness. In turn, risk-taking willingness negatively predicted PSRB. The direct effect of servant leadership on PSRB was also significant. In addition, PSM significantly moderated the positive relationship between work autonomy and PSRB and negatively moderated the relationship between risk-taking willingness and PSRB.

**Conclusion:**

Servant leadership fosters PSRB through both direct and indirect mechanisms. Work autonomy and risk-taking willingness serve as parallel mediators, while PSM qualifies these mediating effects by moderating the second stage of the mediation process.

## 1 Introduction

The “selected candidates” (a special talent recruitment program within the Chinese civil service system) program constitutes a distinctive selection mechanism within China’s civil service system. It primarily targets outstanding university graduates with the aim of cultivating young cadres who possess strong comprehensive qualities and professional capabilities, thereby building a talent pipeline for government agencies at both national and local levels. The program’s emphasis on “high quality” and “professionalism” translates into an expectation of strong public service competence, which requires selected candidates to continuously develop relevant competencies (Zhu et al., 2012). In the contemporary public sector, civil servants shoulder significant responsibilities, a context that can give rise to pro-social rule-breaking (PSRB)—behavior motivated by a proactive desire to maximize organizational interests, even if it necessitates violating formal regulations (He and He, 2019). Previous studies have shown that PSRB does not necessarily produce negative outcomes; rather, it can yield positive effects for individuals and organizations, including enhanced cooperation among stakeholders and improved organizational performance, efficiency, and development (Dahling et al., 2012; Morrison, 2006; Ghosh and Shum, 2019). Moreover, international research has identified several factors influencing PSRB, such as decision-making autonomy, colleagues’ rule-breaking behavior, ethical standards, risk-taking willingness, and public service motivation (PSM) (Khan et al., 2022; Kahari et al., 2017; Vardaman et al., 2014). Given their distinctive status, selected candidates are often entrusted with greater responsibilities and play a vital role in building a service-oriented government. However, most existing studies on this group have focused on policy and theoretical discussion, with relatively few empirical investigations into their PSRB. Against this backdrop, this study takes selected candidates as its focus to empirically examine the internal mechanisms underlying PSRB. In doing so, it seeks to enrich the relevant literature and provide a stronger theoretical foundation for cultivating a high-quality civil service workforce.

## 2 Literature review

Following the conceptualization of Zada et al. (2022), servant leadership is understood as a behavior that empowers subordinates by demonstrating concern for their welfare, prioritizing their career development, and instilling confidence. Servant leadership can be understood through a contemporary five-factor model comprising five critical behavioral pillars. These are: the capacity for Emotional Soothing, the skill of Persuasive Guidance, the principle of Altruism, the application of Leadership Wisdom, and the practice of Social Responsibility (Wang et al., 2025). Previous research has demonstrated that supervisors’ servant leadership positively affects employees’ organizational citizenship behavior, job satisfaction, and job involvement. Since PSRB can be viewed as a form of organizational citizenship behavior, it is similarly influenced by leaders’ attitudes, ranging from encouragement to criticism. For instance, Dahling et al. (2012) developed a generalizable measure of PSRB and found that supervisors’ attitudes could predict increases or decreases in subordinates’ pro-social behaviors. When employees perceive that their leaders will not punish PSRB, they are more likely to engage in it; conversely, the anticipation of punishment reduces this likelihood. According to Social Information Processing Theory (SIPT), individuals’ attitudes and behaviors are shaped by external environmental cues (Ziv and Hadad, 2021). This implies that employees interpret situational factors, such as their leader’s likely response, before deciding whether to undertake risky behaviors like PSRB. Given that leaders hold the authority to sanction rule-breaking, subordinates are particularly inclined to assess their leader’s attitude and adjust their behavior accordingly (Lau and Liden, 2008). Under servant leadership, employees are more likely to believe that their actions will be understood and tolerated, thereby increasing their propensity to engage in PSRB (Khan et al., 2022).

In the context of this study, we extend this logic to civil servants’ perceptions of their supervisors’ servant leadership. We propose that when civil servants perceive their supervisors as practicing servant leadership, they are more likely to view PSRB as justified and beneficial to the organization, and thus more likely to engage in it.

Hypothesis H1: Civil servants’ perception of their supervisors’ servant leadership positively predicts their own PSRB behavior.

Although the motivation underlying PSRB is ultimately positive, employees who violate formal rules still face potential criticism and organizational sanctions. Consequently, an individual’s willingness to take risks—defined here as a work-related disposition reflecting one’s propensity to accept uncertainty and potential negative outcomes in pursuit of goals—is a critical determinant of such behavior (Morrison, 2006). Employees with risk-taking behavior are more inclined to perceive uncertainty as an opportunity and take proactive actions, thereby being more likely to support and commit to organizational change (Jung et al., 2020). In contrast, risk-averse individuals are ascribed traits of indecisiveness and lower agency. These attributions, in turn, are statistically linked to negative expected workplace outcomes, such as a higher perceived likelihood of being downsized, because observers associate passivity with a lack of leadership potential (Fisk and Overton, 2020). Consequently, such individuals are less likely to engage in PSRB, as they tend to overestimate potential adverse outcomes. Prior research on leadership suggests that a risk-taking orientation is associated with transformational leadership and a higher probability of engaging in PSRB (Huang et al., 2014). Extending this logic to subordinates, it is hypothesized that a positive relationship exists between employees’ risk-taking willingness and their engagement in PSRB.

Building on this perspective, we posit that supervisors’ servant leadership can influence subordinates’ risk-taking attitudes. However, we propose that the influence of servant leadership on risk-taking willingness may be culturally contingent. While servant leadership universally emphasizes support and empowerment, its behavioral outcomes can be interpreted differently across cultural contexts. In the Chinese administrative context, which is characterized by high power distance and a strong emphasis on collective harmony and stability, the empowering nature of servant leadership may be interpreted by subordinates not as a signal to pursue personal initiative, but as a responsibility to act with greater caution to maintain stability and avoid bringing unforeseen risks to the collective. Therefore, when perceiving high levels of servant leadership, selected candidates—who are groomed for key roles within a hierarchical system—may experience a heightened sense of responsibility that paradoxically suppresses their willingness to engage in behaviors perceived as personally risky, even for pro-social ends. Servant leaders, by emphasizing support and prioritizing followers’ needs, may thus foster a sense of psychological safety that, in this specific context, leads to more conservative risk appraisals. Importantly, servant leadership does not change a stable personality trait but rather shapes the situational appraisal of risks associated with specific behaviors such as PSRB, an appraisal that is filtered through cultural and institutional norms. Consequently, we propose that risk-taking willingness functions as a mediating mechanism: servant leadership creates a supportive environment that reshapes subordinates’ risk perceptions concerning PSRB, which in turn affects their decisions to engage in it.

Hypothesis H2: Risk-taking willingness mediates the relationship between perceived servant leadership and PSRB behavior.

Work autonomy embodies the freedom and independence employees experience across key facets of their work, most notably in their choice of methods, timing, location, and task sequencing (De Spiegelaere et al., 2016). Prior research consistently shows that autonomy enhances employees’ ability to self-manage their responsibilities, thereby promoting proactive and self-directed behaviors (Ho and Nesbit, 2014; Langfred and Moye, 2004). Servant leadership—characterized by empowerment, trust, and support for follower development—fosters higher levels of perceived work autonomy among subordinates. According to situational leadership theory, leaders who adapt their style to subordinates’ maturity tend to grant greater autonomy, which in turn reinforces mutual trust and responsibility (Shirom et al., 2010). Specifically, by delegating authority, encouraging participation, and demonstrating confidence in their followers’ abilities, servant leaders empower employees to manage their own work processes (Den Hartog and Belschak, 2012).

When employees experience higher levels of autonomy, they develop a stronger sense of ownership and responsibility toward their roles. This heightened sense of responsibility, combined with the discretion to act independently, may encourage them to adopt unconventional approaches—including rule-breaking—when they believe such actions will benefit the organization or the public interest (Hackman and Oldham, 1975; Spreitzer, 1995). For instance, Morrison (2006) empirically demonstrated that work autonomy facilitates PSRB, and Curtis (2010) further confirmed autonomy as a significant predictor of this behavior. Despite these established relationships, the mediating role of work autonomy between servant leadership and PSRB remains underexplored, particularly within the public sector.

We therefore argue that servant leadership enhances employees’ perceived autonomy, which in turn increases their willingness to engage in PSRB when they deem it necessary for achieving broader organizational or societal goals.

Hypothesis H3: Work autonomy mediates the relationship between servant leadership and PSRB behavior.

PSM is defined as an individual’s internal drive to serve the public interest, encompassing a combination of rational, norm-based, and affective motivations (Perry, 1996). While Social Learning Theory (SLT) suggests that leaders can act as role models, potentially influencing subordinates’ PSM (Bandura, 1977), and existing research indicates that servant leadership may enhance followers’ PSM (Tran and Truong, 2021; Weißmüller et al., 2022), the present study considers PSM not as an outcome but as a key boundary condition that shapes the behavioral consequences of psychological states fostered by leadership. To theoretically integrate the role of PSM within our proposed model, we draw upon the motivation–opportunity framework and SIPT (Ziv and Hadad, 2021; Blumberg and Pringle, 1982). This integrated perspective clarifies why PSM is posited to moderate the latter stages of the mediation process—specifically, the relationships between the mediators (work autonomy and risk-taking willingness) and PSRB behavior—rather than the initial link between servant leadership and PSRB. We posit that servant leadership’s influence on subordinates’ immediate psychological experiences, such as their sense of autonomy or risk perceptions, constitutes a relatively proximal psychological process. This process is directly shaped by daily empowering and supportive leader interactions, as suggested by SIPT, wherein the external environment (e.g., leadership behavior) provides social cues that influence individuals’ interpretations of their work situation (Ziv and Hadad, 2021). Accordingly, servant leadership primarily provides the opportunity (through enhanced work autonomy) and alters perceived capacity (by influencing risk perceptions), enabling employees to contemplate non-normative actions such as PSRB.

Conversely, PSM functions as a distal, value-based, and relatively stable motivational orientation that is less susceptible to immediate changes induced by daily leadership interactions (Tran and Truong, 2021; Van Witteloostuijn et al., 2017). Its primary moderating influence is hypothesized to emerge when employees decide whether to act upon the discretion or willingness shaped by leadership. In this sense, PSM serves as a motivational filter that influences how psychological capacities—such as autonomy and risk-taking willingness—are translated into specific pro-social behaviors. For example, employees with high levels of PSM, driven by a commitment to public welfare, are more likely to interpret substantial work autonomy as a mandate to pursue outcomes that benefit the public good, even if this entails bending organizational rules (Morrison, 2006; Weißmüller et al., 2022). Similarly, a high level of PSM can channel risk-taking willingness toward publicly beneficial outcomes, thereby strengthening the relationship between risk-taking willingness and PSRB (Tran and Truong, 2021; Vadera et al., 2013). This reasoning aligns with the motivation–opportunity framework, wherein servant leadership provides the opportunity and capacity, while PSM supplies the essential motivational direction that channels these resources toward PSRB.

This integrated perspective allows us to delineate the distinct roles of these variables in the causal chain. Servant leadership, as an external situational cue, directly shapes more proximal psychological states—namely, the opportunity to act (via enhanced work autonomy) and the appraisal of capacity to act (via altered risk perceptions). These are immediate cognitive and affective responses to the leader’s behavior. In contrast, PSM is a distal, stable, value-based orientation. We posit that its primary function is not to alter these immediately formed perceptions but to govern the translation of these psychological states into actual behavior. It acts as a motivational gatekeeper at the decision-making stage. When an employee contemplates acting upon their autonomy or risk-taking willingness, a high level of PSM provides the necessary motivational impetus to channel these resources toward public-spirited outcomes, even if it involves rule-breaking. Therefore, we hypothesize that PSM’s moderating effect is most salient at the final step—where psychological capacities are converted into pro-social action—rather than at the initial stage of leadership influence or the direct path, which is already strongly driven by the leader’s implicit approval and the established situational cues.

**Figure 1:**
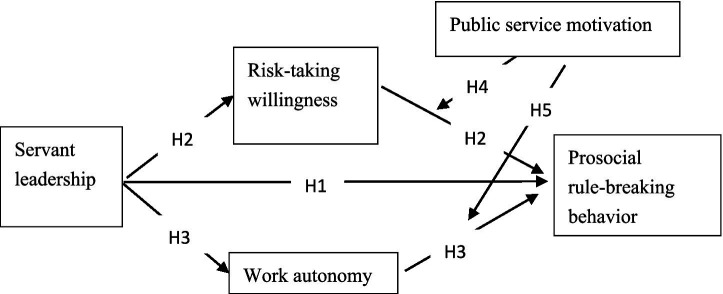
Mediation model diagram.

Accordingly, we propose the following hypotheses concerning the moderated mediation effects: H4: PSM moderates the mediating effect of risk-taking willingness on the relationship between servant leadership and PSRB. Specifically, the mediating effect is stronger when PSM is high. H5: PSM moderates the mediating effect of work autonomy on the relationship between servant leadership and PSRB, such that the mediating effect is stronger when PSM is high. The hypothetical model is shown in Figure 1.

## 3 Research methods

### 3.1 Participants

This study employed a convenience sampling method to collect data via an online survey distributed to selected candidates across China. All variables—including servant leadership, work autonomy, risk-taking willingness, PSM, and PSRB behavior—were measured concurrently using a single online questionnaire. Participation was voluntary, and all respondents provided electronic informed consent. The research protocol was approved by the Ethics Committee of Nanchang University.

A total of 730 responses were collected, of which 51 were excluded due to completion times shorter than 120 s, resulting in 679 valid questionnaires. The demographic characteristics of all participants are presented in Table 1.

**Table 1:** Demographic characteristics of the participants (*N* = 679).

Variable	Category	Number	Percentage (%)
Gender	Male	363	53.5
	Female	316	46.5
Age	20–29 years	426	62.7
	30–39 years	190	28.0
	40–49 years	60	8.8
	50 years and above	3	0.5
Work experience	Less than 1 year	218	32.1
	1–5 years	260	38.3
	6–10 years	153	22.5
	More than 10 years	48	7.1
Education level	Master’s degree or above	271	39.9
	Bachelor’s degree	365	53.8
	College degree or below	43	6.3
Job position	Clerk	333	49.0
	Deputy section chief	188	27.7
	Section chief	96	14.1
	Deputy Department Head	43	6.3
	Department Head or above	19	2.9
Administrative level of department	Township/Street	256	37.7
	County level	266	39.2
	Prefecture-level city	157	23.1

### 3.2 Measures

#### 3.2.1 Servant leadership scale

Servant leadership was measured using a scale developed by Liden et al. (2015), which is one of the most widely used instruments for assessing servant leadership (Eva et al., 2019). The scale comprises seven items, including statements such as “My leader prioritizes my career development” and “My leader emphasizes the importance of giving back to society,” rated on a 5-point Likert scale (1 = strongly disagree, 5 = strongly agree). Servant leadership was assessed by asking participants (the selected candidates) to report their perceptions of their direct supervisor’s leadership behaviors.

#### 3.2.2 Willingness to take risks scale

Risk-taking willingness was measured using a scale developed by Gomez-Mejia and Balkin (1989), consisting of four items such as “I believe one should avoid risks in work at all costs” and “I prefer low-risk, high-security, and stable-paying jobs over high-risk, high-reward jobs,” rated on a 5-point Likert scale (1 = strongly disagree, 5 = strongly agree).

#### 3.2.3 Work autonomy scale

Work autonomy was assessed using a scale developed by Spreitzer (1995), comprising three items such as “I can decide how to do my work” and “I have a fair amount of independence and freedom in my work,” rated on a 5-point Likert scale (1 = strongly disagree, 5 = strongly agree).

#### 3.2.4 PSM scale

PSM was measured using a scale developed by Pandey et al. (2008), consisting of five widely used items recognized as a global standard for assessing PSM. Items include statements such as “Changing society is more important to me than personal achievement” and “Even if it means being ridiculed, I will fight for the rights of others, “covering Perry’s three dimensions: commitment to the public interest, compassion, and self-sacrifice.

#### 3.2.5 PSRB behavior scale

PSRB behavior was assessed using a scale developed by Dahling et al. (2012), consisting of 13 items, such as “I would break organizational rules to work more efficiently” and “I would break organizational rules to provide the best help to the people,” rated on a 5-point Likert scale. PSRB was measured via participant self-reports of their own behaviors.

#### 3.2.6 Common method bias (CMB)

Common method bias refers to systematic variance that is unrelated to the constructs being measured, potentially compromising measurement validity. Although CMB is common in empirical research, it can be controlled through procedural and statistical approaches. In this study, procedural controls included geographically separated data collection and the inclusion of both positively and reverse-coded items in the questionnaire. Additionally, Harman’s single-factor test was employed to statistically assess the presence of common method bias.

### 3.3 Statistical analysis

Data analysis was conducted using AMOS 24.0, SPSS 26.0, and the PROCESS 3.5 macro (Models 1, 4, and 14) developed by Hayes. The analyses aimed to examine the current status, influencing factors, and underlying mechanisms of the selected candidates’ behaviors.

## 4 Results

### 4.1 Homology bias test

To examine potential common method bias in the questionnaire data, Harman’s single-factor test was conducted. Principal component analysis extracted five factors with eigenvalues greater than 1. The first factor accounted for 39.42% of the total variance, which is below the critical threshold of 40%, indicating that common method bias is unlikely to pose a significant threat in this study.

### 4.2 Descriptive statistics and correlation matrix of the variables

The analyses revealed significant positive correlations among servant leadership, work autonomy, PSM, and PSRB behavior. In contrast, servant leadership, work autonomy, PSM, PSRB behavior, and risk-taking willingness showed clear negative correlations. Detailed results are presented in Table 2.

**Table 2:** Means, standard deviations, and correlation coefficients of the variables.

Variables	M	SD	1	2	3	4	5
1. Servant leadership	3.83	0.69	1				
2. Risk-taking willingness	2.73	0.62	−0.218**	1			
3. Work autonomy	2.86	0.64	0.636**	−0.167**	1		
4. PSM	5.13	0.73	0.463**	−0.222**	0.347**	1	
5. PSRB behavior	3.54	0.93	0.608**	−0.222**	0.599**	0.283**	1

*** = *p* < 0.001, ** = *p* < 0.01, * = *p* < 0.05. The significance level of the *p*-values is consistent with that in the table below.

### 4.3 Regression analysis

After obtaining the correlation results for the four variables—servant leadership, risk-taking willingness, work autonomy, and PSRB behavior—further validation of their predictive relationships was conducted. Hierarchical regression analyses were performed using SPSS 26.0 to examine the effects of servant leadership, risk-taking willingness, and work autonomy on PSRB behavior. The results are presented in Table 3. Risk-taking willingness (*β* = −0.140, *t* = −3.323, *p* < 0.001) showed a significant negative predictive effect on the PSRB behavior of the selected candidates, whereas work autonomy (*β* = 0.426, *t* = 8.174, *p* < 0.001) exhibited a significant positive predictive effect. The regression model for PSRB behavior produced an *R*^2^ of 0.504 and an adjusted *R*^2^ of 0.498, indicating that the independent variables explained 50.4% of the variance in the dependent variable. The model demonstrated good overall fit, with an *F*-value of 85.069 (*p* < 0.001) in the ANOVA, suggesting that the constructed multiple linear regression model is statistically significant.

**Table 3:** Regression model of the effects of PSRB behavior.

Model: PSRB behavior		Unstandardized coefficients	Std. error	Standardized coefficients	*t*	Sig (*p*)	95.0% Confidence interval for *B*		Collinearity statistics	
		*β*		Beta			Lower	Upper	Tolerance	VIF
(Constant)		0.700	0.229	—	3.058	0.002	0.251	1.150	—	—
Independent variable	Servant leadership	0.449***	0.049	0.332	9.102	<0.001	0.352	0.546	0.557	1.794
Mediating variable	Risk-taking willingness	−0.140***	0.042	−0.093	−3.323	<0.001	−0.222	−0.057	0.941	1.063
	Work autonomy	0.426***	0.052	0.295	8.174	<0.001	0.324	0.529	0.568	1.759
Control variable	Age	0.058	0.064	0.042	0.907	0.365	−0.067	0.183	0.350	2.857
	Length of service	0.142**	0.046	0.139	3.069	0.002	0.051	0.234	0.359	2.784
	Educational background	−0.132**	0.041	−0.102	−3.254	0.001	−0.212	−0.052	0.759	1.317
	Position	0.047	0.035	0.054	1.341	0.180	−0.022	0.117	0.453	2.210
	Department	0.072	0.037	0.059	1.926	0.055	−0.001	0.144	0.791	1.264
Model fit	*R* ^2^	0.504								
	Adjusted *R*^2^	0.498								
	*F*	85.069***								

Hierarchical regression analyses were conducted using SPSS 26.0 to examine the effect of servant leadership on risk-taking willingness. The results are presented in Table 4. Servant leadership (*β* = −0.185, *t* = −5.057, *p* < 0.001) had a significant negative predictive effect on risk-taking willingness. The regression model yielded an *R*^2^ of 0.058 and an adjusted *R*^2^ of 0.049, indicating that servant leadership accounted for 5.8% of the variance in risk-taking willingness. The model demonstrated a moderate fit, which may reflect the cultural preference for stability commonly observed in China. The ANOVA results indicated an *F*-value of 6.858 (*p* < 0.001), suggesting that the constructed multiple linear regression model is statistically significant.

**Table 4:** Regression model of the effects of risk-taking willingness.

Model: risk-taking willingness		Unstandardized coefficients	Std. error	Standardized coefficients	*t*	Sig (*p*)	95.0% Confidence interval for *B*		Collinearity statistics	
		*β*		Beta			Lower	Upper	Tolerance	VIF
(Constant)		3.400	0.160	—	21.293	0.000	3.087	3.714	—	—
Independent variable	Servant leadership	−0.186***	0.037	−0.206	−5.057	<0.001	−0.258	−0.114	0.843	0.186
Control variable	Age	−0.095	0.058	−0.103	−1.626	0.104	−0.209	0.020	0.351	0.845
	Length of service	0.021	0.042	0.031	0.498	0.619	−0.062	0.104	0.365	0.739
	Educational background	−0.016	0.037	−0.018	−0.425	0.671	−0.089	0.057	0.761	0.313
	Position	0.044	0.032	0.076	1.372	0.170	−0.019	0.108	0.454	0.203
	Department	0.052	0.034	0.064	1.515	0.130	−0.015	0.118	0.795	0.258
Model fit	*R* ^2^	0.058								
	Adjusted *R*^2^	0.049								
	*F*	6.858***								

Hierarchical regression analyses were conducted using SPSS 26.0 to examine the effect of servant leadership on work autonomy. The results are presented in Table 5. Servant leadership (*β* = 0.536, *t* = 18.046, *p* < 0.001) had a significant positive predictive effect on work autonomy. The regression model for work autonomy yielded an *R*^2^ of 0.431 and an adjusted *R*^2^ of 0.426, indicating that servant leadership explained 43.1% of the variance in work autonomy. The model demonstrated good fit, with an *F*-value of 84.732 (*p* < 0.001) in the ANOVA, suggesting that the constructed multiple linear regression model is statistically significant.

**Table 5:** Regression model of the effects of work autonomy.

Model: Work autonomy		Unstandardized coefficients	Std. error	Standardized coefficients	*t*	Sig (*p*)	95.0% Confidence interval for *B*		Collinearity Statistics	
		*β*		Beta			Lower	Upper	tolerance	IF
(Constant)		0.688	0.129	—	5.339	0.000	0.435	0.941	—	—
Independent variable	Servant leadership	0.536***	0.030	0.572	18.046	<0.001	0.478	0.594	0.843	0.186
Control variable	Age	0.023	0.047	0.024	0.488	0.626	−0.069	0.115	0.351	2.845
	Length of service	0.112**	0.034	0.158	3.284	0.001	0.045	0.179	0.365	2.739
	Educational background	−0.038	0.030	−0.043	−1.280	0.201	−0.097	0.021	0.761	1.313
	Position	−0.002	0.026	−0.004	−0.096	0.924	−0.054	0.049	0.454	2.203
	Department	−0.024	0.027	−0.028	−0.859	0.391	−0.077	0.030	0.795	1.258
Model fit	*R* ^2^	0.431								
	Adjusted *R*^2^	0.426								
	*F*	84.732***								

### 4.4 Testing the moderating effects

The mediating effects of work autonomy and risk-taking willingness were tested using Model 4 of Hayes’ PROCESS 3.5 macro. After controlling for potential confounding variables, we examined whether work autonomy and risk-taking willingness predicted PSRB behavior. The results indicated that both risk-taking willingness [mediating effect = 0.03, SE = 0.01, 95% CI = (0.01–0.06)] and work autonomy [mediating effect = 0.23, SE = 0.03, 95% CI = (0.17–0.30)] had significant mediating effects. These findings suggest that work autonomy and risk-taking willingness jointly mediate the relationship between servant leadership and PSRB behavior. As shown in Table 6, the mediating effect of work autonomy accounted for 23.2% of the total effect, whereas the mediating effect of risk-taking willingness accounted for 3% of the total effect. In summary, hypotheses H1, H2, and H3 were all supported.

**Table 6:** Mediating effects of work autonomy and risk-taking willingness.

Path	Effect type	Effect	SE	t	Sig (*p*)	95% Confidence interval	
						LLCI	ULCI
Servant leadership- > Risk-taking willingness- > PSRB behavior	Total	0.703	0.042	16.601	<0.001	0.620	0.787
	Direct	0.675	0.043	15.762	<0.001	0.591	0.759
	Indirect	0.029	0.012	2.375	<0.001	0.009	0.056
Servant leadership- > Work autonomy- > PSRB behavior	Total	0.703	0.042	16.601	<0.001	0.620	0.787
	Direct	0.471	0.049	9.576	<0.001	0.375	0.568
	Indirect	0.232	0.034	6.741	<0.001	0.165	0.300

### 4.5 Test of moderated mediation effects

After controlling for potential confounding variables, a moderated mediation analysis was conducted using Models 1 and 14 of Hayes’ PROCESS 3.5 macro to examine whether PSM moderates the relationships between work autonomy and PSRB behavior, as well as between risk-taking willingness and PSRB behavior.

**Figure 2:**
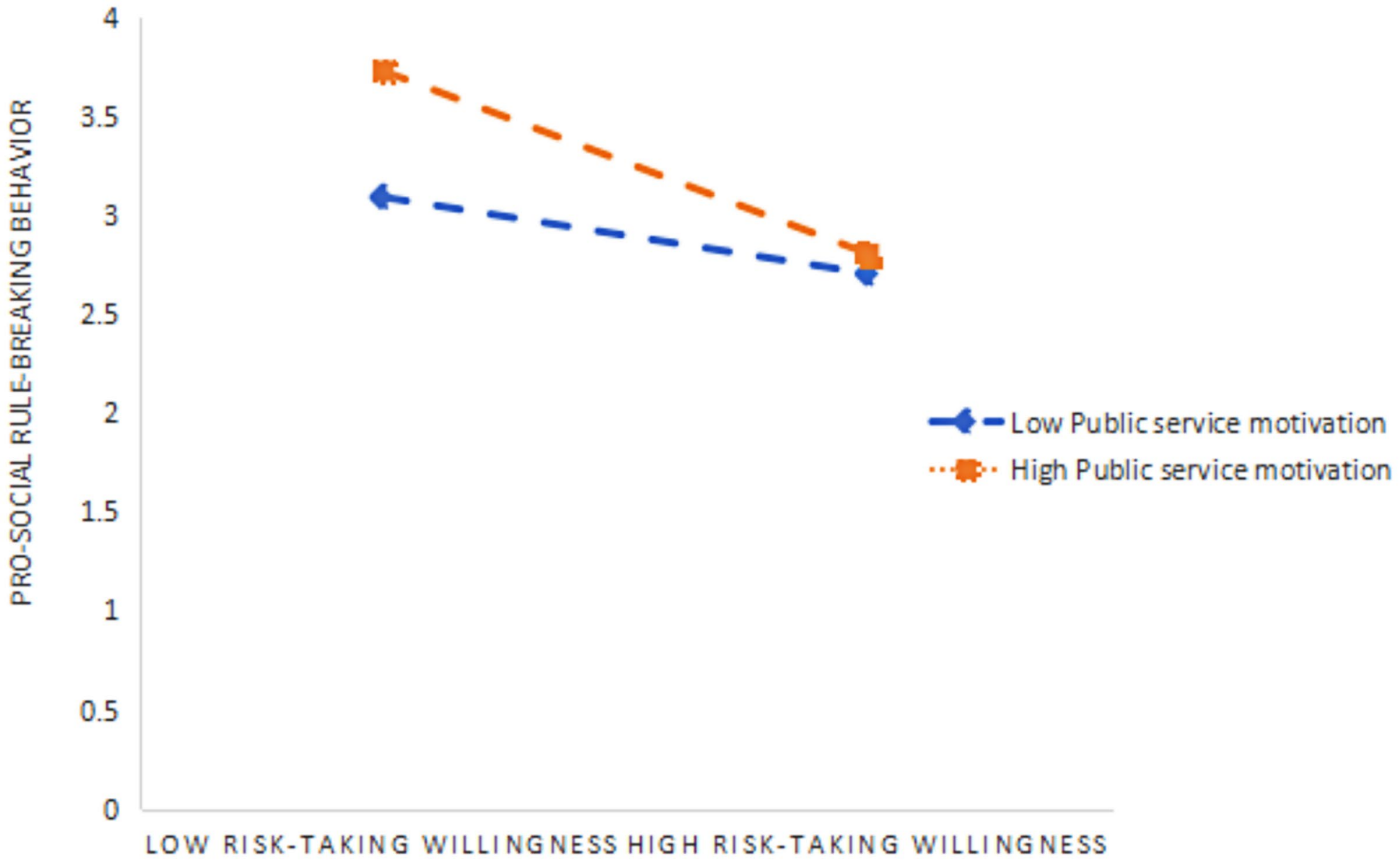
Slope diagram of the moderating effect of PSM on PSRB behavior in terms of Risk-taking willingness.

**Figure 3:**
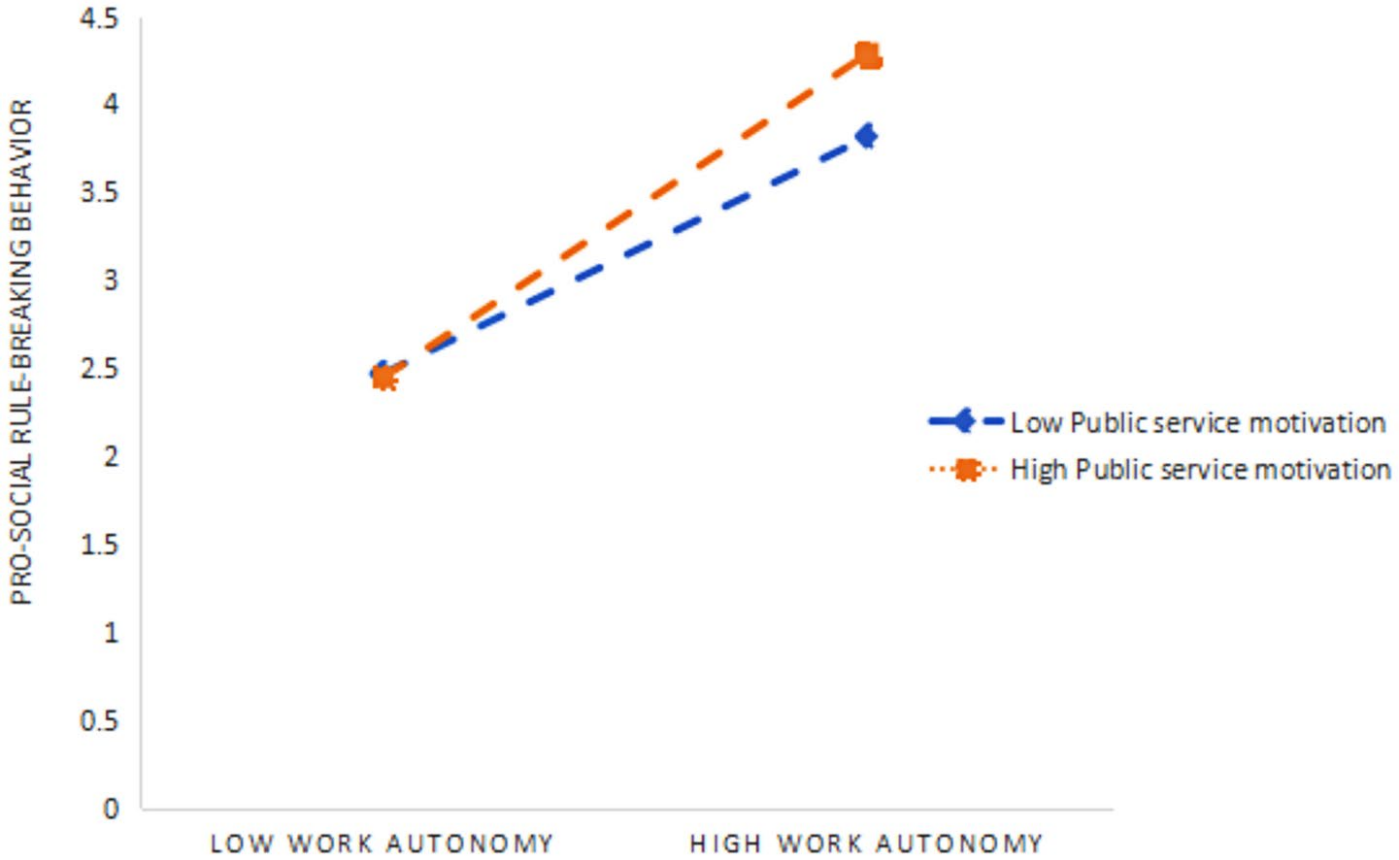
Slope of the moderating effect of PSM on PSRB behavior in risk-taking willingness.

**Figure 4:**
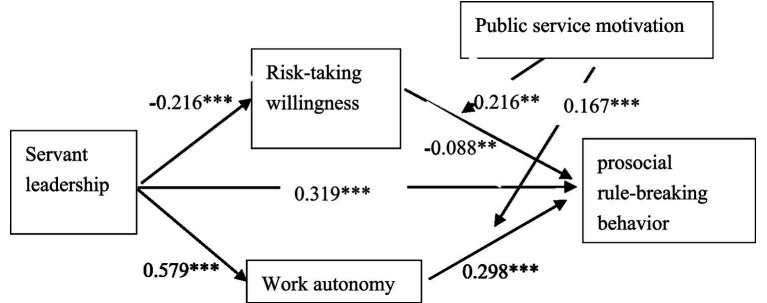
Research model diagram.

As presented in Tables 7, 8, PSM significantly and negatively moderated the relationship between risk-taking willingness and PSRB behavior (*β* = −0.26, *p* < 0.001), while simultaneously exerting a significant positive moderating effect on the relationship between work autonomy and PSRB behavior (*β* = 0.25, *p* < 0.001). To aid interpretation, simple slope tests were conducted. As shown in Table 9, when PSM was at a low level, the mediating effect of risk-taking willingness was non-significant. However, at higher levels of PSM, the mediating effect of risk-taking willingness became significant, with an effect size of 0.047, indicating that the mediating role of risk-taking willingness is contingent on PSM (Figure 2). Similarly, as illustrated in Table 10, when PSM was low, the effect size of work autonomy was 0.177. When PSM increased to a higher level, the mediating effect of work autonomy significantly increased to 0.317 (Figure 3), demonstrating that the mediating role of work autonomy is also moderated by PSM. In conclusion, both hypotheses H4 and H5 were supported. The overall research model of this study is illustrated in Figure 4.

**Table 7:** Testing the moderating effect of PSM on risk-taking willingness on PSRB behavior.

Model		Coeff (*β*)	SE	t	Sig (*p*)	95% Confidence interval	
						LLCI	ULCI
(Constant)		2.896	0.135	21.446	0	2.631	3.161
Independent variable	Risk-taking willingness	−0.193***	0.05	−3.885	<0.001	−0.29	−0.095
Moderator variable	PSM	0.369***	0.046	8.055	<0.001	0.279	0.459
Int (Risk-taking willingness × PSM)		−0.269***	0.062	−4.368	<0.001	−0.39	−0.148
Control variable	Age	0.128	0.074	1.722	0.086	−0.018	0.274
	Length of service	0.351***	0.052	6.685	<0.001	0.248	0.454
	Educational background	−0.146**	0.048	−3.075	0.002	−0.24	−0.053
	Position	−0.016	0.041	−0.397	0.691	−0.097	0.065
	Department	0.049	0.044	1.135	0.257	−0.036	0.135
model fit	*R* ^2^	0.320					
	△*R*^2^	0.019					
	*F*	39.425***					

**Table 8:** Test of the moderating effect of PSM on PSRB behavior by work autonomy.

Model		Coeff (*β*)	SE	*t*	Sig (*p*)	95% Confidence interval	
						LLCI	ULCI
(Constant)		3.145	0.121	25.987	0	2.908	3.383
Independent variable	Work autonomy	0.675***	0.047	14.219	<0.001	0.582	0.768
Moderator variable	PSM	0.226***	0.042	5.397	<0.001	0.144	0.309
Int (Work autonomy × PSM)		0.248***	0.045	5.473	<0.001	0.159	0.337
Control variable	Age	0.092	0.066	1.389	0.165	−0.038	0.221
	Length of service	0.200***	0.048	4.169	<0.001	0.106	0.295
	Educational background	−0.128**	0.042	−3.005	0.003	−0.211	−0.044
	Position	0.014	0.037	0.377	0.706	−0.058	0.086
	Department	0.047	0.039	1.223	0.222	−0.029	0.123
Model fit	*R* ^2^	0.462					
	△*R*^2^	0.024					
	*F*	71.827***					

**Table 9:** Moderated mediation effect of PSM on the relationship between risk-taking willingness, servant leadership, and PSRB behavior.

Model	PSM	Coeff (*β*)	SE	95% Confidence interval	
				LLCI	ULCI
Servant Leadership → Risk-taking Willingness → PSRB Behavior	Low (M − 1SD)	0.004	0.017	−0.032	0.036
	High (M + 1SD)	0.047	0.02	0.015	0.094
	Difference (High–Low)	0.043	0.029	−0.004	0.109
	Moderated mediation index	0.030	0.020	−0.003	0.075

**Table 10:** Moderated mediation effect of PSM on the relationship between work autonomy, servant leadership, and PSRB behavior.

Model	PSM	Coeff (*β*)	SE	95% Confidence interval	
				LLCI	ULCI
Servant Leadership → Work autonomy → PSRB Behavior	Low (M − 1SD)	0.177	0.04	0.089	0.248
	High (M + 1SD)	0.317	0.046	0.236	0.417
	Difference (High–Low)	0.140	0.051	0.067	0.265
	Moderated mediation index	0.096	0.035	0.046	0.182

## 5 Discussion

Based on the data from this study, the perceived level of servant leadership among the sampled public servants was above the scale midpoint, suggesting that leaders in government departments are gradually adapting their leadership styles in response to evolving circumstances and organizational contexts. These leaders increasingly emphasize attention to subordinates, respect their contributions, and support their professional development. Work autonomy was slightly above average, indicating that Chinese civil servants enjoy a moderate degree of discretion and freedom in managing their work. Notably, civil servants with 6–10 years of experience reported the highest levels of perceived autonomy, followed by those with more than 10 years of experience. This pattern may reflect the general association between longer tenure and higher organizational rank, which typically confers greater decision-making authority. Risk-taking willingness was generally slightly below average, suggesting a relatively low willingness among Chinese civil servants to engage in risky behaviors at work. This may be influenced by historically prevalent authoritative leadership styles. In contrast, PSM was generally high, indicating a strong intrinsic motivation to serve the public, potentially rooted in cultural and societal values. PSRB behavior was slightly below average, with low consistency across respondents, suggesting that engagement in such behavior is context-dependent and shaped by individual experiences and perceptions.

Consistent with our hypotheses, the results indicate that servant leadership is positively associated with PSRB, suggesting that higher levels of perceived servant leadership correspond to a greater likelihood of engaging in PSRB. This observation aligns with prior research (Zhu and Wang, 2014; Lu et al., 2017). In management contexts, leaders can act as significant external situational factors that influence subordinates’ behaviors and organizational outcomes (Khan et al., 2022)^.^ According to Social Information Processing Theory, external situational cues can shape individuals’ attitudes, motivations, and behaviors. In this study, servant leadership—as perceived by subordinates—may provide positive reinforcement for PSRB, creating expectations that such behaviors will be understood or supported by leaders. Consequently, subordinates may be more inclined to engage in PSRB.

It is important to emphasize that these interpretations are based on cross-sectional data, and the observed relationships are consistent with, but do not confirm, the hypothesized mediating or causal mechanisms. While the findings suggest a potential pathway through which servant leadership may influence PSRB, definitive causal claims cannot be made. Moreover, the construct of servant leadership reflects individual-level perceptions of immediate supervisors’ behaviors, situating the analysis and its interpretations at the subordinate level.

Furthermore, servant leadership was positively associated with PSRB, with work autonomy partially accounting for this relationship. Previous studies have seldom examined the internal mediation mechanisms linking leadership style and PSRB. The limited research that has explored work autonomy as a mediator—for example, between ethical leadership and PSRB—found a partial mediating effect. Similarly, Yang (2019) investigated work autonomy as a mediator between servant leadership and change-oriented behavior, reporting a partial mediation. In the public sector, where work processes and procedures are highly emphasized, leadership care, support, and understanding are particularly influential for subordinates. Servant leadership involves entrusting employees with important responsibilities and demonstrating high levels of trust, which encourages them to address problems or make key work decisions independently, fostering positive leader–subordinate interactions. PSRB, as a form of rule-breaking behavior, requires both leadership support and a certain degree of autonomy. By promoting work autonomy, servant leadership may facilitate PSRB in ways that aim to maximize organizational outcomes and provide junior officials with opportunities to navigate the “many tasks, little authority” dilemma, potentially enhancing overall efficiency and effectiveness.

Additionally, risk-taking willingness was found to partially account for the relationship between servant leadership and PSRB. It is critical to clarify the nature of this indirect effect. Although servant leadership negatively predicted risk-taking willingness (path a = −0.21, *p* < 0.001), which in turn negatively predicted PSRB (path b = −0.09, *p* < 0.001), the resulting indirect effect was positive (a × b = 0.03). This seemingly counterintuitive finding—that a negative pathway yields a positive mediation—can be explained within the specific cultural context of this study. We posit that servant leadership, by creating a supportive environment, reduces subordinates’ perceptions of the risks associated with PSRB. This diminished risk perception, paradoxically fostered by a lower general willingness to take risks, creates a “psychological safety zone” that indirectly facilitates rather than inhibits employees’ engagement in PSRB for the greater good. This finding contrasts with the more universalistic view that empowering leadership invariably promotes risk-taking. This divergence underscores the culturally contingent nature of leadership effects. As theorized, within China’s administrative context—characterized by high power distance and a collectivist emphasis on stability—the trust and empowerment inherent in servant leadership may be interpreted by subordinates as a responsibility to exercise caution and maintain organizational harmony. This heightened sense of collective responsibility, paradoxically fostered by a supportive leader, appears to suppress individual risk-taking willingness, even for pro-social ends. Decision theory suggests that individuals evaluate and select among options with varying levels of risk, and proactive leadership styles have been identified as key situational factors influencing such decision-making (Hameduddin and Engbers, 2022)^.^ Accordingly, both leadership style and risk-taking willingness can be considered factors shaping employee decisions, which in turn may relate to PSRB. Consequently, the direct effect of servant leadership on PSRB is stronger than its indirect effect through risk-taking willingness. This pattern highlights that in this specific cultural context, direct leadership cues dominate over an individual’s innate risk-taking willingness in influencing PSRB.

It is important to note, however, that the observed associations are based on cross-sectional data. While the results are consistent with mediation, they do not provide definitive evidence of causal mechanisms. Even when candidates exhibit high risk-taking willingness, cultural and organizational norms in China—which emphasize a pragmatic and cautious work approach—may lead individuals to self-regulate, perceiving risky behaviors as impulsive or socially undesirable. Under such considerations, employees may moderate or avoid risk-taking, potentially limiting engagement in PSRB. Moreover, high risk-taking willingness could reduce psychological security, further constraining such behavior (Xu and Zhu, 2017). Given the substantial authority held by leaders, subordinates are likely to weigh leadership responses heavily when contemplating PSRB. Consequently, the direct association of servant leadership with PSRB appears stronger than the indirect influence via risk-taking willingness, a pattern that aligns with our theoretical expectations but should be interpreted with caution due to the cross-sectional nature of the data.

As hypothesized, PSM was observed to moderate the relationships between work autonomy and risk-taking willingness and their respective mediating roles in the association between servant leadership and PSRB. PSM, as an intrinsic altruistic motivation, represents a distinctive form of public sector employee motivation, characterized by goal-oriented behaviors aimed at contributing to society, cooperating with others, and selflessly dedicating oneself to public interests. Previous research indicates that servant leadership can positively influence employees’ PSM, supporting its development (Khan et al., 2022; Tran and Truong, 2021). Furthermore, Weißmüller et al. (2022) reported that higher levels of PSM are associated with increased engagement in PSRB, suggesting that other-oriented PSM may encourage employees to engage in PSRB. Importantly, the observed moderation effects should be interpreted as conditional associations consistent with the hypothesized pathways rather than definitive causal mechanisms. In the present study, when PSM is low, the association between risk-taking willingness and PSRB is non-significant, as is the mediating effect of risk-taking willingness. As PSM increases, the association between risk-taking willingness and PSRB becomes significant and is substantially strengthened. Work autonomy, by contrast, maintains a consistent positive association with PSRB across all levels of PSM, with its mediating effect also remaining significant and increasing progressively with higher PSM levels.

The results suggest that PSM may differentially influence the indirect pathways linking servant leadership to PSRB. These findings validate our theoretical model, wherein PSM acts not as a mediator but as a motivational gatekeeper at the decision-making stage, determining how psychological capacities are behaviorally enacted. First, the observed negative correlations among risk-taking willingness, PSRB, and PSM may have contributed to the moderation patterns. Second, employees with higher PSM, guided by intrinsic altruistic motives and sensitivity to social evaluation, may attenuate risk-taking behaviors driven by self-interest, thereby weakening the association between risk-taking willingness and PSRB. Third, work autonomy, PSM, and PSRB appear to share a common orientation toward promoting organizational development and collective interests. Consequently, higher PSM may enhance the relationship between work autonomy and PSRB and strengthen its mediating role, potentially creating a reinforcing cycle that aligns individual motivation with organizational goals. It is essential to reiterate that these findings are based on cross-sectional data. While the patterns observed are consistent with moderated mediation, causal inferences cannot be established, and future longitudinal or experimental studies are needed to confirm these mechanisms.

## 6 Conclusion and implications

This study offers two key theoretical refinements. First, it challenges the universalistic assumption of servant leadership’s effect on risk-taking by demonstrating a culturally specific pathway. Our findings suggest that in contexts prioritizing stability and hierarchy, empowerment may foster a sense of responsibility that encourages caution over daring, thereby refining the nomological network of servant leadership. Second, it clarifies the functional role of PSM within a leadership-process model. By employing the motivation-opportunity framework, we position PSM not as a mediator or a moderator of the initial leadership impact, but as a critical motivational catalyst that determines whether psychologically empowered employees will ultimately enact PSRB, thereby specifying the boundary conditions of the psychological mechanisms linking leadership to behavior.

Building on these theoretical insights, our findings offer several practical implications for public sector management, while recognizing the limitations of cross-sectional data. First, at the leadership development level, organizations may benefit from emphasizing and cultivating a servant leadership style. Training managers to empower, trust, and support subordinates can enhance employees’ perceived work autonomy and foster an organizational climate that permits appropriate risk-taking for the public good. Such an environment may encourage greater engagement in PSRB and contribute to organizational effectiveness, though causal confirmation requires further longitudinal or experimental investigation. Second, in terms of organizational system design, while adherence to core rules remains essential, public organizations might reflect on overly rigid processes. Granting employees reasonable work autonomy and establishing clear “error-tolerance mechanisms” can provide systemic safeguards that enable flexible responses in emergency or special situations, reducing inaction stemming from fear of accountability. Third, human resource management practices may consider the role of PSM. PSM can serve as a valuable criterion in personnel recruitment and selection, and performance appraisal systems could be designed to recognize and reward innovative behaviors that, while deviating from conventional rules, achieve positive public value. Such practices may reinforce employees’ altruistic motivations and alignment with organizational goals. It is important to note that this study was conducted within the specific context of China’s civil service system, characterized by strong hierarchical norms, collectivist values, and an emphasis on political loyalty. These contextual factors may influence how servant leadership is perceived and how PSRB is enacted. Consequently, caution should be exercised when generalizing these findings to other cultural or administrative settings. Future research is encouraged to replicate this model in diverse public sector environments to evaluate its cross-cultural applicability.

Although this study systematically examined the relationships among servant leadership, work autonomy, risk-taking willingness, PSM, and PSRB, several limitations should be acknowledged. First, the cross-sectional design precludes strong causal claims regarding the mediating roles of work autonomy and risk-taking willingness. While the analyses provide statistical evidence consistent with mediation, longitudinal or experimental studies are needed to validate the causal pathways. Second, the sample was limited to cadres in grassroots government departments, representing only a subset of the civil service population. Therefore, generalization of the findings to the broader civil service should be approached with caution. Finally, all data were collected via self-reported questionnaires, which may be susceptible to social desirability bias. Future research could combine implicit situational experiments with questionnaire measures to enhance the robustness and validity of the findings.

The present study provides evidence that Chinese civil servants exhibit moderately high levels of servant leadership, work autonomy, and PSM, whereas their risk-taking willingness and engagement in PSRB are generally moderate to low. Servant leadership is positively associated with PSRB, with both risk-taking willingness and work autonomy serving as partial mediators. Moreover, PSM moderates the relationships between these mediators and PSRB, underscoring its role as an important boundary condition that shapes the behavioral outcomes of leadership in public sector contexts. While these findings are consistent with the hypothesized model, causal interpretations are tentative due to the cross-sectional design, and future research is needed to confirm the observed associations.

## Data Availability

The datasets presented in this article are not readily available because the data that support the findings of this study are available from Nanchang University, but restrictions apply to the availability of these data, which were used under license for the current study and so are not publicly available. The data are, however, available from the authors upon reasonable request and with the permission of Nanchang University. Requests to access the datasets should be directed to 394138156@qq.com.
